# Pancreaticoduodenectomy for preservation of fat-replaced pancreatic body and tail tissue in a patient with solid pseudopapillary neoplasm: a case report

**DOI:** 10.1186/s40792-020-00894-x

**Published:** 2020-06-15

**Authors:** Toru Sakurai, Yuichi Nagakawa, Chie Takishita, Hiroaki Osakabe, Hitoe Nishino, Masanori Akashi, Naoto Okazaki, Kenta Suzuki, Kenji Katsumata, Akihiko Tsuchida

**Affiliations:** Department of Gastrointestinal and Pediatric Surgery, Tokyo Medical University, 6-7-1 Nishishinjuku, Shinjuku-ku, Tokyo, 160-0023 Japan

**Keywords:** Pancreatoduodenectomy, Total pancreatectomy, Solid pseudopapillary neoplasm, Fat-replaced pancreatic tissue, Agenesis/Aplasia/Dysplasia/Hypoplasia of pancreatic body and tail

## Abstract

**Background:**

There is no standard surgical method for treating pancreatic head tumors with fat replacement of the pancreatic body and tail. Total pancreatectomy procedures are usually performed to excise pancreatic head tumors and lead to endocrine function loss and subsequent development of diabetes. We present a rare case where the adipose tissue was preserved during pancreaticoduodenectomy in a patient with a solid pseudopapillary neoplasm and fat-replaced pancreatic body and tail.

**Case presentation:**

Contrast-enhanced computed tomography scans of a 43-year-old man revealed a tumor measuring approximately 3 cm in size with calcification in the pancreatic head. Magnetic resonance cholangiopancreatography showed that the pancreatic ducts in the body and tail were completely disrupted. Furthermore, endoscopic ultrasonography showed no pancreatic parenchyma in the body and tail of the pancreas, with disruption in the main pancreatic duct. Endoscopic ultrasonography-guided fine-needle aspiration led to the final pathological diagnosis of a solid pseudopapillary neoplasm, and laparoscopic total pancreatectomy was performed. However, intraoperative findings indicated that the tumor was located in the pancreatic head. Pancreatic parenchyma was not observed in the pancreatic body or tail, as it had been completely replaced with adipose tissue. Nevertheless, the shape of the pancreas was identifiable. Therefore, pancreaticoduodenectomy was performed to transect parenchyma at the pancreatic neck, while preserving the adipose tissue present in the pancreatic body. The main pancreatic duct could not be identified at the cut surface. Therefore, we performed modified Blumgart-style pancreaticojejunostomy to cover the cut end instead of reconstructing the pancreatic duct. The patient was discharged on postoperative day 12 without complications and is being followed-up as an outpatient. His fasting blood sugar and hemoglobin A1c levels according to the National Glycohemoglobin Standardization Program reports were within normal limits, indicating that the endocrine function (insulin secretion ability) was preserved during the 1.5 years following surgery.

**Conclusions:**

In patients with pancreatic head tumors, pancreaticoduodenectomy that preserves fat-replaced pancreatic body and tail tissues can preserve postoperative endocrine function.

## Background

The most appropriate surgical method for patients with a pancreatic head tumor and fat-replaced pancreatic body and tail has not been established [1–14]. Essentially, total pancreatectomy (TP) is performed to excise the pancreatic head tumor, which results in subsequent loss of endocrine function and development of diabetes. Here, we report on a novel case in which adipose tissue was preserved during pancreaticoduodenectomy (PD) in a patient with a solid pseudopapillary neoplasm (SPN) and fat-replaced pancreatic body and tail. Preservation of the adipose tissue enabled retention of postoperative endocrine function and insulin secretion ability. Considering the novelty of our case, we also conducted a literature review of current evidence to gain insights into the procedural approaches in similar cases.

## Case presentation

**Fig. 1:**
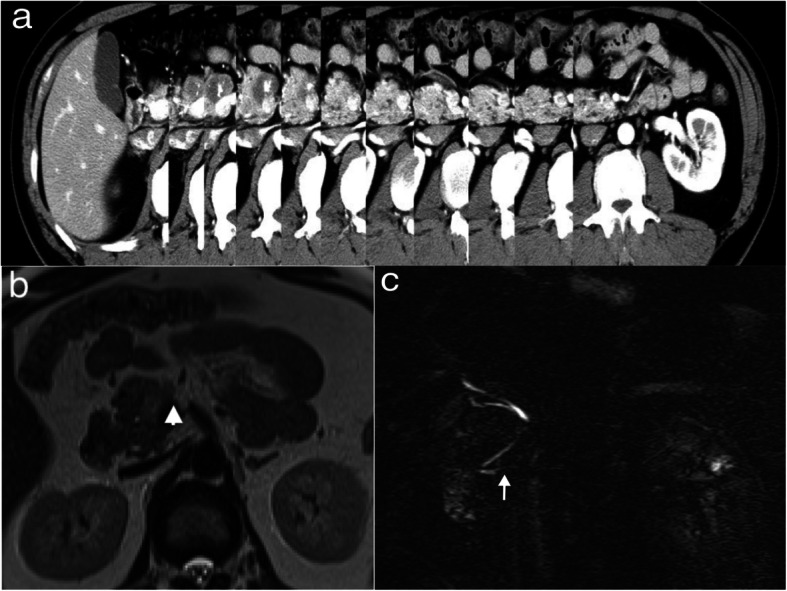
Abdominal contrast-enhanced computed tomography and magnetic resonance imaging findings*.***a** Abdominal contrast-enhanced computed tomography scan showing a mass measuring 30 mm in diameter with calcification, which projected forward in the pancreatic head without the body and tail. **b** T2-weighted magnetic resonance imaging scans showing a high signal intensity in the body and tail of the pancreas (arrowhead). **c** Magnetic resonance cholangiopancreatography image showing complete disruption of the pancreatic duct of the body and tail (arrow)

The medical examination of a 43-year-old man revealed calcification in the pancreatic head. He was referred to the Department of Gastroenterology for examination. No symptoms were evident, and his medical history was unremarkable. Contrast-enhanced computed tomography (CT) scans revealed a tumor (approximate size, 3 cm) with calcification in the pancreatic head. The pancreatic duct and parenchymal tissue were not observed on the caudal side of the portal vein. There were no liver metastases or enlargement of the surrounding lymph nodes. T1-weighted magnetic resonance imaging (MRI) scans showed low signal intensity, while T2-weighted MRI scans showed a reasonably high signal intensity in the pancreatic head. Furthermore, T2-weighted MRI scans indicated a high signal intensity in the pancreatic body and tail. Magnetic resonance cholangiopancreatography (MRCP) revealed that the pancreatic ducts of the pancreatic body and tail were completely disrupted (Fig. 1). Endoscopic ultrasonography (EUS) showed a low echoic mass with calcification, while contrast-enhanced harmonic EUS (CH-EUS) (Sonazoid, GE Healthcare, Oslo, Norway) revealed stark contrasts and hypovascularity in the tumor. EUS-guided fine-needle aspiration (EUS-FNA) was performed. Subsequently, the patient was diagnosed with a SPN.

**Fig. 2:**
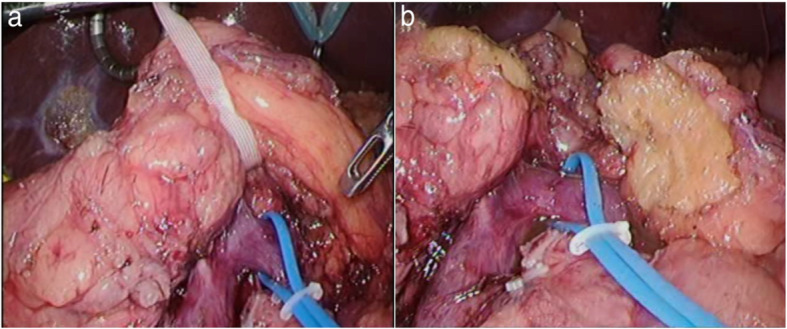
Surgical findings. **a** Caudal pancreatic tissue is replaced by adipose tissue. **b** Cut end of the pancreatic body replaced by adipose tissue

**Fig. 3:**
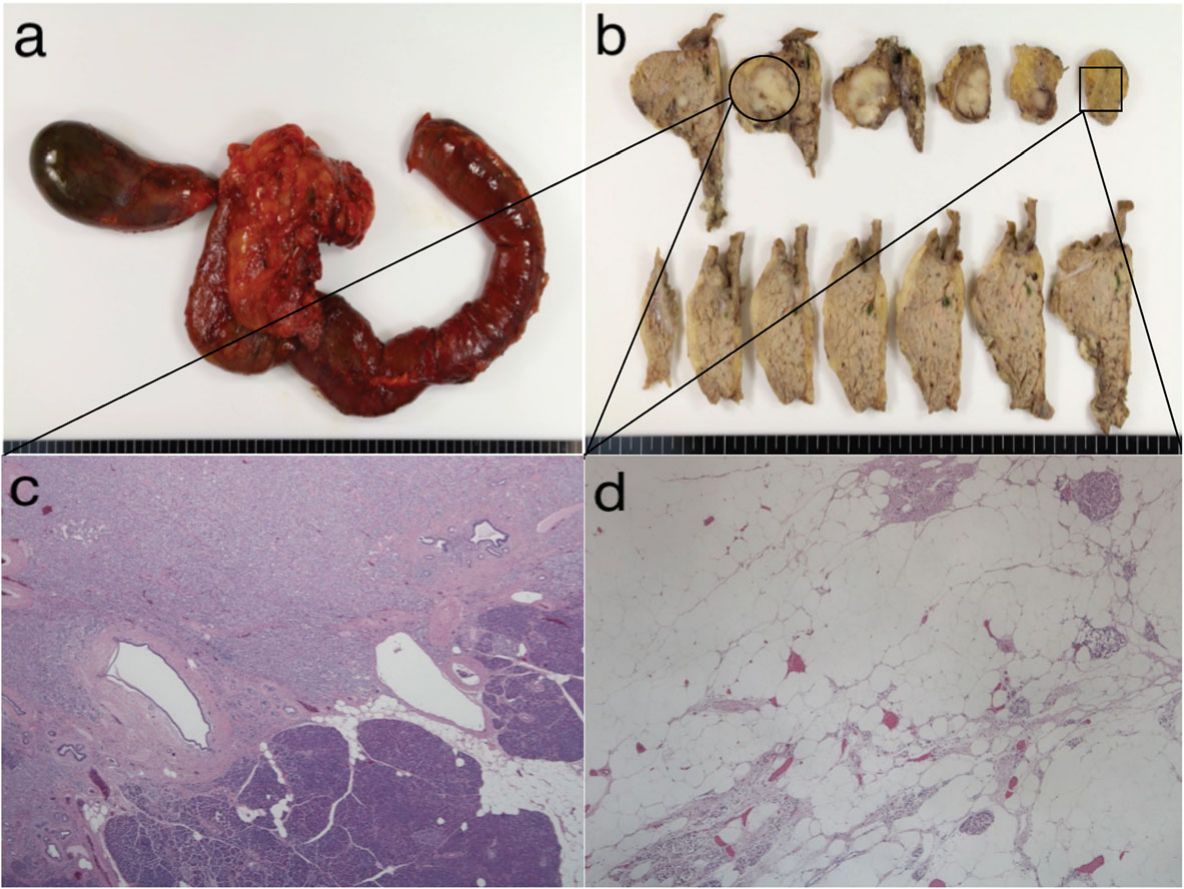
Surgical specimens and histopathological findings. **a** Extracted specimen. **b** Examination of the resected specimen revealing a white nodular tumor extending to the body of the pancreas. The tumor contained calcified and hemorrhagic areas. **c** Histopathological findings (hematoxylin and eosin staining, × 20). **d** The pancreatic tissue is replaced by adipose tissue, and only a few Langerhans islets are observed (hematoxylin and eosin staining, × 40)

Initially, we planned to perform a laparoscopic TP as intraoperative findings indicated that the tumor was present in the pancreatic head. Additionally, there was no pancreatic parenchyma in the pancreatic body, while that in the tail was completely replaced by adipose tissue. However, the shape of the pancreas could be identified. Based on these findings, a PD was performed on the left side of the portal vein (Fig. 2). As we found that the tumor had invaded the portal vein, we switched to open surgery. The pancreatic duct was not reconstructed, as the main pancreatic duct could not be identified at the cut surface. Therefore, only a modified Blumgart-style pancreaticojejunostomy was performed to cover the cut end. The surgery lasted for 425 min, and the volume of blood loss was 500 mL. Histopathological findings indicated that the pancreatic tissue was replaced by adipose tissue, and only Langerhans islets were observed (Fig. 3).

The patient was discharged on postoperative day 12 without complications. He is being followed-up 1.5 years after the surgery. His fasting blood sugar and hemoglobin A1c (HbA1c) levels according to the National Glycohemoglobin Standardization Program (NGSP) reports were within the normal limits (HbA1c NGSP, preoperatively, 5.5%; 3 months postoperatively, 5.7%), indicating that endocrine function (insulin secretion ability) was preserved. He experienced diarrhea three times a day after the surgery; however, his nutritional status was maintained with oral administration of pancrelipase. His preoperative body weight and serum albumin levels were 87.6 kg and 4.6 g/dL, respectively, and by 3 months postoperatively, these were 81.4 kg and 4.6 g/dL, respectively.

## Conclusions

Studies have reported that approximately 60% of patients with congenital aplasia in the body and tail of the pancreas develop diabetic complications [15]. Takahashi et al. [16] reported that atrophy and vitrification of the Langerhans islets were noted in autopsy cases of early-onset congenital aplasia in the body and tail of the pancreas, whereas an increased number of Langerhans islets and hypertrophy were found in some late-onset cases. Thus, diabetes mellitus in congenital aplasia of the body and tail of the pancreas is not caused by a single factor, but rather by the lack of Langerhans islets and several other acquired factors [16].

An electronic search in the Japan Medical Abstracts Society database (Ichushi web) using the phrases “agenesis of dorsal pancreas,” “aplasia of the pancreatic body and tail,” “dysplasia of the pancreatic body and tail,” and “hypoplasia of pancreatic body and tail” (excluding proceedings) yielded 91 case reports published since 1990. In 14 [1–14] of the 91 reports, the patients had undergone either TP or PD (Table 1). In TP cases, six patients developed diabetes before surgery and two patients developed diabetes after surgery. However, in PD cases (*n* = 6), patients did not develop diabetes at all. The loss of pancreatic tissue in the body and tail of the pancreas was noted in most patients who underwent TP; however, adipose tissue replacement of pancreatic tissue was not observed in any of these patients. Patients who underwent PD had no remnant pancreatic tissue, but did have adipose tissue, with the remaining Langerhans islets having replaced the lost pancreatic tissue.

**Table 1:** Reported cases of agenesis of body and tail of the pancreas undergoing total pancreatectomy or pancreaticoduodenectomy

No.	Year	Author	Age	Sex	Disease	Operation	Pancreatic tissue^a^	Replaced fat tissue^b^	Langerhans islets^c^	Preoperative diabetes	Postoperative diabetes
1	1991	Fukata et al. [1]	54	F	Pancreatic head cancer	TP	Absent	Absent	Absent	None	Developed
2	1996	Furuichi et al. [2]	49	M	Pancreatic head cancer	TP	Absent	Absent	Absent	None	Developed
3	1997	Kishinaka et al. [3]	59	M	Malignant pancreas head endocrine tumor	TP	Absent	Present	Present	Developed	Developed
4	1999	Kuroki et al. [4]	75	M	Pancreatic cancer	PD	Present	Present	Unknown	None	None
5	2001	Nio et al. [5]	72	F	Pancreatic head cancer	PD	Absent	Present	Unknown	None	None
6	2002	Kanazumi et al. [6]	47	F	Lower bile duct carcinoma	TP	Absent	Absent	Absent	Developed	Developed
7	2002	Teshigawara et al. [7]	70	M	Chronic pancreatitis with pseudotumor	TP	Absent	Absent	Absent	Developed	Developed
8	2004	Yamada et al. [8]	73	F	Pancreatic head cancer	PD	Absent	Present	Present	None	None
9	2004	Funato et al. [9]	43	F	Nonfunctioning malignant islet cell tumor	PD	Absent	Present	Absent	None	None
10	2005	Tobita et al. [10]	57	F	Carcinoma of the ampulla of Vater complicated by von Recklinghausen’s disease	PD	Hypoplasia	Absent	Unknown	None	Not developed
11	2009	Ijichi et al. [11]	62	F	Malignant solid pseudopapillary pancreas tumor	TP (PD)	Absent	Present	Unknown	None	Not developed
12	2013	Oki et al. [12]	65	M	Pancreatic cancer	TP	Absent	Absent	Unknown	Developed	Developed
13	2014	Ito et al. [13]	79	F	Carcinoma of the ampulla of Vater	TP	Absent	Absent	Unknown	Developed	Developed
14	2018	Suzuki et al. [14]	50	F	Pancreatic neuroendocrine tumor	TP	Absent	Absent	Unknown	Developed	Developed
15	2019	Our case	43	M	Pancreatic head SPN	PD	Absent	Present	Present	None	Not developed

*TP* total pancreatectomy, *PD* pancreaticoduodenectomy

^a^Pancreatic tissue of cut end

^b^Replaced fat tissue of pancreatic body and tail

^c^Langerhans islets of cut end

These results suggested that the Langerhans islets were retained in cases with adipose tissue replacement, which preserved endocrine function; thus, the patients did not develop diabetes. Therefore, we believed that performing PD with retention of fat-replaced pancreatic body and tail tissues in patients with pancreatic head tumors can preserve postoperative endocrine function. In addition, we performed a literature search for the reported procedures aimed at preserving adipose tissue in the pancreatic body and tail after PD. Tobita et al. [10] reported a patient with hypoplasia rather than agenesis of the dorsal pancreas. Furthermore, they found a main pancreatic duct requiring pancreaticojejunostomy. However, postoperative pancreatic juice leakage was observed. In our case, complete fat replacement of the pancreatic body and tail preserved the shape of the pancreas, but the main pancreatic duct could not be identified. Therefore, a modified Blumgart-style pancreaticojejunostomy was performed to prevent pancreatic juice leakage.

In addition, our literature review did not reveal any cases with anastomosis in the presence of fat-replaced pancreatic body and tail tissues, as performed in our case. Furthermore, postoperative pancreatic juice leakage was not observed in our case. These observations suggest that in cases where the remaining pancreatic tissue is completely replaced by adipose tissue and the main pancreatic duct is not identifiable, it may not be necessary to perform pancreaticojejunostomy.

In conclusion, in patients with the pancreatic head tumors, pancreaticoduodenectomy that preserves fat-replaced pancreatic body and tail tissues can preserve postoperative endocrine function.

## Data Availability

Data sharing is not applicable to this article as no datasets were generated or analyzed during the current study.

## Abbreviations

TP: Total pancreatectomy
PD: Pancreatoduodenectomy
SPN: Solid pseudopapillary neoplasm
CT: Computed tomography
MRI: Magnetic resonance imaging
MRCP: Magnetic resonance cholangiopancreatography
EUS: Endoscopic ultrasonography
EUS-FNA: EUS-guided fine-needle aspiration
CH-EUS: Contrast-enhanced harmonic EUS
LPD: Laparoscopic pancreaticoduodenectomy
NGSP: National Glycohemoglobin Standardization Program
